# *PTEN* deletion drives acute myeloid leukemia resistance to MEK inhibitors

**DOI:** 10.18632/oncotarget.27206

**Published:** 2019-10-08

**Authors:** Amanda M. Smith, Christine R.C. Zhang, Alexandre S. Cristino, John P. Grady, J. Lynn Fink, Andrew S. Moore

**Affiliations:** ^1^ The University of Queensland Diamantina Institute, The University of Queensland, Woolloongabba, Australia; ^2^ Oncology Services Group, Queensland Children’s Hospital, South Brisbane, Australia; ^3^ Child Health Research Centre, The University of Queensland, South Brisbane, Australia; ^4^ Current address: Washington University in Saint Louis, Saint Louis, Missouri, United States of America; ^5^ Current address: Garvan Institute of Medical Research, Darlinghurst, Australia; ^6^ Current address: Griffith Institute for Drug Discovery, Brisbane Innovation Park, Nathan, Australia

**Keywords:** myeloid leukemia, therapy, drug resistance, hematological malignancies, gene mutation

## Abstract

Kinases such as MEK are attractive targets for novel therapy in cancer, including acute myeloid leukaemia (AML). Acquired and inherent resistance to kinase inhibitors, however, is becoming an increasingly important challenge for the clinical success of such therapeutics, and often arises from mutations in the drug-binding domain of the target kinase. To identify possible causes of resistance to MEK inhibition, we generated a model of resistance by long-term treatment of AML cells with AZD6244 (selumetinib). Remarkably, resistance to MEK inhibition was due to acquired *PTEN* haploinsufficiency, rather than mutation of MEK. Resistance via this mechanism was confirmed using CRISPR/Cas9 technology targeting exon 5 of *PTEN*. While *PTEN* loss has been previously implicated in resistance to a number of other therapeutic agents, this is the first time that it has been shown directly and in AML.

## INTRODUCTION

Pediatric acute myeloid leukemia (AML) causes a disproportionate number of childhood cancer deaths [1, 2]. The peak incidence of childhood AML occurs in children under 4 years (1.1 per 100,000; 1982–2007) [2, 3]. The event-free survival for childhood AML is only 50 to 60% despite complete remissions being achieved in approximately 90% of children with front-line therapy [4]. Furthermore, improvements in overall survival for childhood AML over the past 30 years can be largely attributed to more intensive use of conventional cytotoxics and improved supportive care [5]. Conventional therapy for pediatric AML parallels strategies that have been used in adults and is based on intensive use of cytarabine (cytosine arabinoside, Ara-C), etoposide and anthracyclines [6]. For relapsed patients, most therapeutic regimens include further cytarabine in combination with other agents [7]. The effectiveness of chemotherapy is limited by not only acute toxicity, but also late effects such as an increased risk of secondary malignancy and cardiotoxicity [8, 9]. These chemotherapy-associated side effects are of particular concern in children given that treatment occurs during growth and development. Therefore, there is a desperate need for more efficacious, yet less toxic, therapeutic strategies to improve outcomes for pediatric AML patients.

Substantial evidence supports an essential role of Ras/Raf/MEK/ERK1/2 kinases in tumourigenesis [10]. Constitutive activity of the Ras/Raf/MEK/ERK1/2 signaling cascade has been reported in a wide variety of liquid and solid cancer types including AML, acute lymphoblastic leukemia (ALL), breast, melanoma and prostate cancer [11]. Inappropriate or prolonged activation of the Ras/Raf/MEK/ERK1/2 pathway have been suspected to lead to altered gene expression and contribute to cancer and chemotherapy resistance [12]. Therefore, small molecule inhibition of kinases within the pathway, such as MEK, has been investigated for their therapeutic potential [13, 14].

Several MEK inhibitors have shown promising pre-clinical activity in adult AML, with a number of compounds currently being evaluated in phase I/II clinical trials, including MEK162, GSK1120212 and AS703026 [15–19]. However, the frequent development of resistance to kinase inhibitors, such as imatinib and sorafenib, through a variety of mechanisms such as acquisition of additional mutations within the kinase domains of the target proteins, leads us to an important question: will MEK inhibitor utility in AML be limited by rapid selection and expansion of subclones that either express or develop intrinsically resistant mutations in the pathway or that lack dependence on MEK signaling for growth and survival? To answer this question, we established an *in vitro* model of MEK inhibitor resistance using THP-1 cells, incubated in increasing concentrations of selumetinib (clinical trial NCT00588809; completed), to identify the mechanism (s) that may lead to resistance in AML patients. We identified a partial deletion of the *tumour suppressor phosphatase and tensin homolog* (*PTEN*) as a clinically relevant genomic alteration in 100% of replicate resistance cell lines that we developed. We also confirmed that disruption of the PTEN catalytic core motif domain was the mechanism of resistance using CRISPR-mediated deletion of *PTEN* exon 5, a region which contributes to the catalytic activity of this tumour suppressor [20]. Given the observation that *PTEN* deletion is a mechanism for MEKi resistance in solid tumours [21, 22], and that deletion of *PTEN* in AML is sufficient to confer resistance, we propose that this event can be used as a biomarker of MEK inhibitor resistance in AML. This is also the first report of *PTEN* deletion as a mechanism of small molecule inhibitor resistance in AML.

## RESULTS

### MEK inhibitors reduce proliferation in AML cells *in vitro*

There is a considerable amount of *in vitro*, *in vivo* and clinical data investigating the utility of MEK inhibitors to treat adult AML [13, 14]. However, it is not known if MEK inhibitors are efficacious in treating pediatric AML. Therefore, we firstly interrogated whether pediatric AML-derived cell lines were sensitive to MEK inhibitors *in vitro.* We used a panel of seven MEK inhibitors and tested them in 11 cell lines including six pediatric and five adult AMLs encompassing the most prevalent cytogenetic and molecular features found in patients. We found varying sensitivity across all compounds tested in a proliferation assay. However, the majority exhibited nanomolar IC_50_s across most cell lines (Table 1). Interestingly, one pediatric AML cell line CMK and one adult AML cell line HEL showed overt resistance to MEK inhibitors suggesting an intrinsic resistance that does not require drug exposure or selection pressure to develop.

**Table 1:** MEK inhibition reduces the proliferation of AML cells

Pedatric	Cell line	Cytogenetics	Sequence Features	PD0325901	AZD6244	MEK162	GSK1120212	CI1040	TAK733	AS703026	Ara-C	Daunorubicin	Etoposide
	**MV-4-11**	MLL-AF4	FLT3-ITD	0.17**	0.41*	0.33**	0.003***	6.9	0.089**	0.41**	1.6	**0.048**	**0.056**
	**THP-1**	MLL-AF9	TP53(R174fs*3), NRAS (G12D)	0.19***	0.3**	0.43**	0.08***	2.2*	0.23***	0.23***	6.7	0.122	1.9
	**CMK**	Myeloid leukaemia associated with Down Syndrome. Complex	TP53(D49H and M133K), CDKN2A (M1_*157del), JAK3 (A572V), GATA1(E2fs*37)	>20	>20	>20	>20	>20	>20	>20	0.48	0.076	4.5
	**AML-193**	**Complex karyotype with 4% polyploidy**	**NRAS (G13V)**	0.24*	2.9	2.1	0.14*	**4.8**	0.25*	0.42*	2.6	0.057	3.4
	**Kasumi-1**	t (8;21)(q22; q22)	RUNX1-CBFA2T1; c-KIT mut (N822K), TP53 (R248Q)	0.2	1.7	1.1	0 004	6.5	0.051	0.31	0.15	0.028	0.53
	**M-07e**	t (11;21)(p11; p13)	CREBBP (Q2208H)	0.11	1.1	0.34	0.006	4.8	0.038	0.08	0.033	0.0069	0.18
Adult	**ME1**	CBFB-MYH11		0.003	0.022	0.037	0.00004	0.55	0.005	0.08	0.41	0.36	18
	**HL-60**	**MLL-AF6**	CDKN2A (R80* and P135L), NRAS (Q61L), t (6,11)(q27; q23)	0.001	0.12	0.02	0.001	0.73	0.001	0.007	0.64	0.02	1.3
	**ML-2**	KMT2A-AFDN	CREBBP (L1090*) NOTCH1(P2514fs*4), KRAS (A146T), CDKN2A (M1*157del)	0.004	0.066	0.047	0.001	0.68	0.01	0.02	0.069	0.011	0.16
	**HEL**	Hypertriploid with 2.3% polyploidy	JAK2 (V617F), TP53 (M133K), CDKN2A (M1_*157del)	>20	>20	>20	>20	>20	>20	>20	0.085	0.12	1.6
	**OCI-AML3**	Hyperdiploid (+1, +5, +8, der (1) t(1;18)(p11; q11), i (5p), del (13)(q13q21), dup (17)(q21q25))	DNMT3A (R882C), NPM1 (W288fs*12)	0.008	0.07	0.04	9.20E-05	1.1	0.004	0.02	>20	0.019	0.47
**Median all**				**0.11**	**0.3**	**0.33**	**0.003**	**2.2**	**0.038**	**0.08**	**0.445**	**0.048**	**1.3**

IC50 is defined as the concentration of drug that reduces cell viability by 50%, calculated by non-linear regression. Median all is calculated excluding those values >20 uM where an exact IC50 could not be established.

Previous studies have shown that the MEKi PD0325901 can inhibit cell cycle progression in the OCI-AML3 cell line [23] and GSK1120212 induces cytostatic, rather than cytotoxic, effects in particular cytogenetic backgrounds in a variety of cancer cell lines [16]. Therefore, it is possible that MEKi-associated anti-proliferative effects observed in Table 1 are a consequence of MEKi-induced cell cycle arrest. To test this hypothesis, we interrogated cell cycles in the AML cells after a 96 h treatment with MEKi (0.01, 0.1, 1 μM) (Supplementary Figure 1). We observed a range of cell cycle responses, with most cell lines exhibiting reduced S/G2/M populations in response to MEKi.

### MEKi synergize with chemotherapeutic compounds

The basis for poor clinical response in AML patients is attributed to the development of resistance to current chemotherapeutic protocols. One mechanism of resistance is thought to be due to hyperactivation of the MAPK signalling pathway owing to the production of reactive oxygen species, a by-product of chemotherapy [11]. Therefore, we hypothesized that inhibition of MAPK signalling in addition to treatment with chemotherapy would sensitize cells to death and potentially prevent the development of resistance. To investigate the potential for synergistic therapeutic effect between MEKi and the chemotherapeutic compounds Ara-C, etoposide and daunorubicin, four pediatric AML cell lines were co-treated for 96 h at fixed drug ratios (Table 2). The combination index (CI; quantitative measure of the extent of drug interaction) was calculated for the ED50 (effective dose) using Chou-Talalay analysis [24]. AZD6244 was the most synergistic when combined with all cytotoxics examined across all four cell lines with ED50 CI of <0.01-0.96. Combinations with other MEKi were variable (Table 2). However, it appeared that etoposide in combination with MEKi trended towards antagonism (>1.1). Interestingly, co-treatment of CMK cells (which are overtly resistant to MEKi monotherapy) with either cytarabine or daunorubicin sensitized the cells to the inhibition of proliferation. These data suggest that combination therapy of MEKi with cytotoxics that are currently in use clinically for pediatric AML is a potential strategy to sensitize AML cells to growth inhibition and cell death, even in the cells that are resistant to MEKi monotherapy.

**Table 2:** Combination index between MEK inhibitors and conventional chemotherapeutic compounds

Cell line	Drug A	Drug B	Combination index	
		Cytarabine	0.34	**+++**
	AS703026	Daunorubicin	0.31	**+++**
		Etoposide	>1.1	**-**
		Cytarabine	0.49	**+++**
**CMK**	AZD6244	Daunorubicin	<0.01	**+++**
		Etoposide	0.6	**+++**
		Cytarabine	0.32	**+++**
	GSK1120212	Daunorubicin	0.14	**+++**
		Etoposide	>1.1	**-**
		Cytarabine	>1.1	**-**
	AS703026	Daunorubicin	>1.1	**-**
		Etoposide	>1.1	**-**
		Cytarabine	0.24	**+++**
**Kasumi1**	AZD6244	Daunorubicin	0.96	±
		Etoposide	0.24	**+++**
		Cytarabine	>1.1	**-**
	GSK1120212	Daunorubicin	>1.1	**-**
		Etoposide	>1.1	**-**
		Cytarabine	0.85	**++**
	AS703026	Daunorubicin	0.95	±
		Etoposide	1.1	±
		Cytarabine	0.42	**+++**
**MV-4-11**	AZD6244	Daunorubicin	0.47	**+++**
		Etoposide	0.52	**+++**
		Cytarabine	>1.1	**-**
	GSK1120212	Daunorubicin	0.82	**++**
		Etoposide	>1.1	**-**
		Cytarabine	0.64	**+++**
	AS703026	Daunorubicin	0.67	**+++**
		Etoposide	>1.1	**-**
		Cytarabine	0.36	**+++**
**THP-1**	AZD6244	Daunorubicin	0.43	**+++**
		Etoposide	0.51	**+++**
		Cytarabine	>1.1	**-**
	GSK1120212	Daunorubicin	>1.1	**-**
		Etoposide	>1.1	**-**

Combination index was calculated from the ED50 using Chou-Talalay analysis in the CalcuSyn software; -, antagonism (>1.1); ±, additive (0.9–1.1); ++, moderate synergism (0.7–0.9); +++, synergism (0.3–0.7).

### Acquired PTEN haploinsufficiency contributes to MEKi resistance

Resistance to small molecule inhibitors is a recognized clinical problem across a range of cancers. It has become evident that surveillance for resistance is imperative to detect early relapse in patients and understanding the mechanisms and biomarkers for resistance is key. Therefore, we developed eight THP-1 cell lines independently derived to have resistance against AZD6244, subsequently referred to as TR, to investigate the how cells acquire resistance to MEKi.

**Figure 1:**
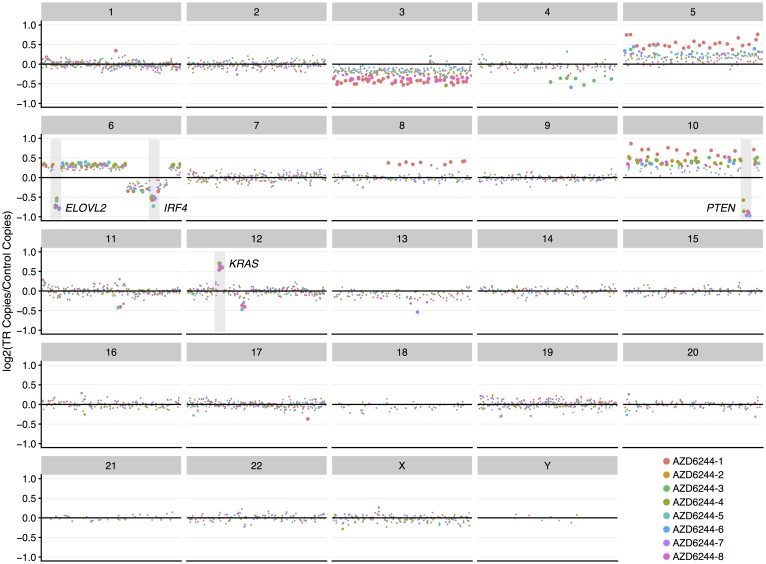
Copy number changes acquired during generation of MEKi-resistant phenotype. Copy number variation analysis of all TR populations revealed amplification and deletion events common to all samples which are known to be associated with cancer and/or drug resistance. These events include deletion of *PTEN*, *IRF4*, and *ELOLV2* and amplification of *KRAS*. TR copy changes are shown relative to DMSO controls. Each panel describes copy changes for the chromosome indicated in the title bar. Circle size indicates the statistical significance of the difference in gene copies (Bonferroni-adjusted p-values); large circles represent p < 0.001, medium circles represent 0.001 < p < 0.05; and small circles represent p ≥ 0.05. Circle colour represents replicates.

We hypothesized that TR cells acquired somatic mutations in MEK-related genes which contribute to MEKi resistance [25]. In order to characterize these acquired mutations, we performed targeted exome sequencing on THP-1 cells grown in culture with DMSO only (control) (n=8) and on the TR (n=8) cells. A total of 239 single nucleotide variants (SNVs) were identified (Supplementary File 1). However, no SNVs were recurrent across all resistant lines suggesting they were not the drivers of resistance. The most commonly occurring SNV was a missense mutation in *NTRK*2 (chr9:87570296, in 4 of 8 resistant lines) (Supplementary Figure 2). However, the low variant allele frequencies (range: 12%-19%) suggest that this mutation is likely subclonal and does not contribute significantly to MEKi resistance. In contrast to small number of SNVs, copy number changes were found in 835 genes including a single, clonal copy loss of *PTEN* in all 8 resistant samples (Supplementary File 2). We also observed subclonal copy gain of *KRAS* in all 8 samples, as well as subclonal copy loss of both *ELOVL2* and *IRF4* (Figure 1), all of which have been identified in tumours although the effect of these copy number losses in AML is unknown. Interestingly, *PTEN* exons appeared to be affected differently. For example, exon 2 appeared to be unaffected in the resistant lines, whereas exons 5 to 9, which encode the motif contributing to the catalytic phosphatase activity of PTEN [20], seemed to be consistently lost.

**Figure 2:**
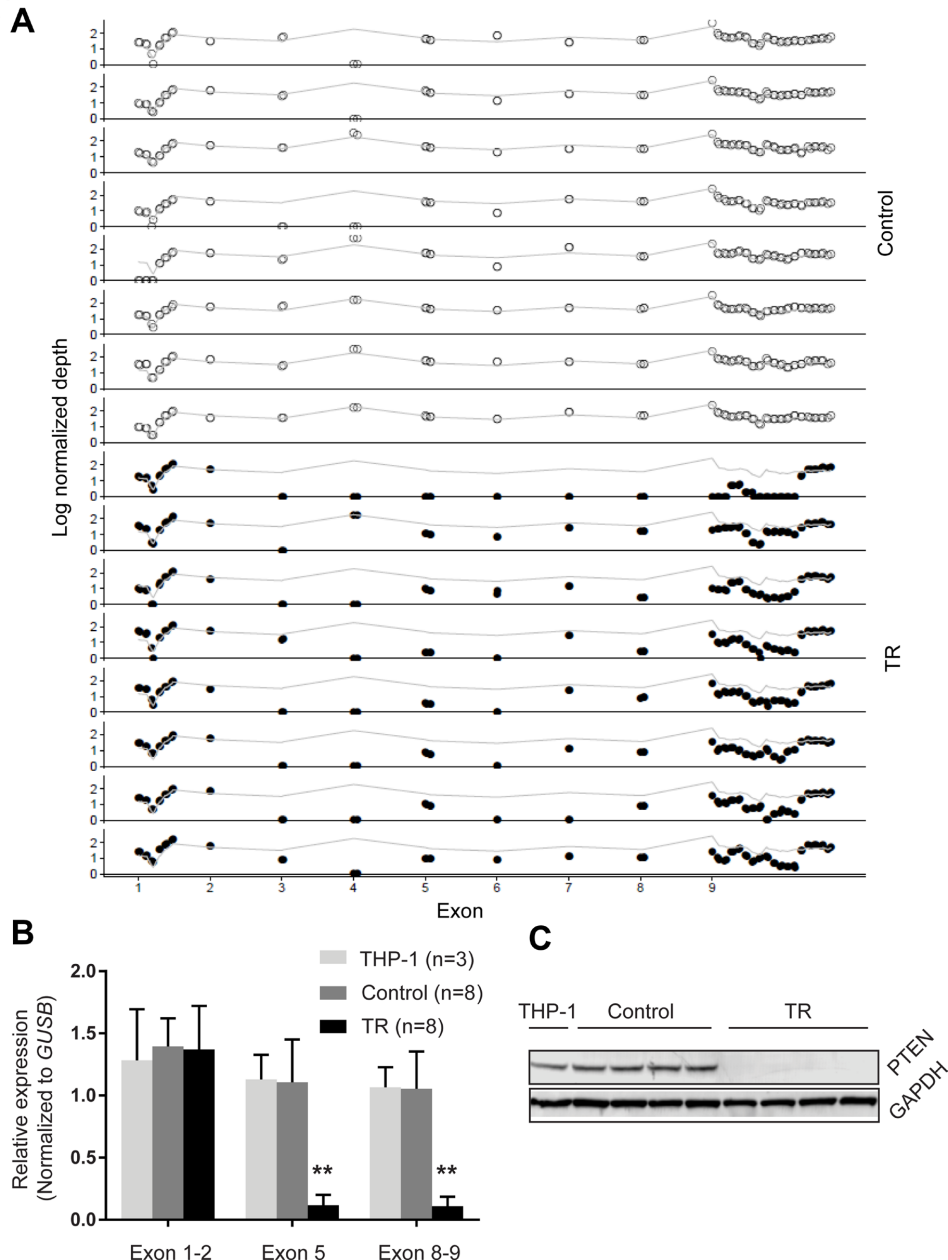
*PTEN* loss in TR cells. (**A**) Copy number changes of *PTEN* exons. Normalized depth (log) of each probe targeting *PTEN* (indicated by o and •) was obtained by targeted exome sequencing. Grey line indicates the average depth of the entire locus. Dots above and below the grey line indicate gain and loss in copy numbers, respectively, at the targeted locus. (**B**) qPCR analysis of *PTEN* expression. The transcriptional levels of the exon 1–2, exon 5 and exon 8–9 of *PTEN* were determined by qPCR and normalized to *GUSB*. (**C**) Representative western blot of PTEN levels in the parental cells (lane 1), control cells (2–5) and resistant cells (6-9). GAPDH (37 kD) was used as a loading control for each blot. Data are presented as mean and standard deviation. **p < 0.01 (*t-*test, two-tailed).

To interrogate the functional impact of the deletion of large regions of *PTEN* as observed in the TR cells, we investigated whether genomic copy losses were reflected in transcriptional levels. Due to the importance of exons 5, 8 and 9 in the function and stability of PTEN, we quantified the expression of these regions, as well as the region encompassing exons 1 and 2, which was unaffected in resistance cells, as a control. Our results showed that the exons 1 and 2 had similar transcriptional levels across TR, control and parental (THP-1) lines, while we observed a significant decrease in the expression of the exons 5, 8 and 9 in the TR cells (Figure 2B). Moreover, PTEN levels, measured by immunoblot with an antibody specific to the C-terminal epitope, paralleled our qPCR results where PTEN levels were barely detected in the resistant cells, but were consistent and detectable in both parental cells and control cells (Figure 2C). These results show that the alterations in *PTEN* were perpetuated at both the transcript and protein levels.

### Introduction of truncated PTEN in parental THP-1 cells results in MEK inhibitor resistance

**Figure 3:**
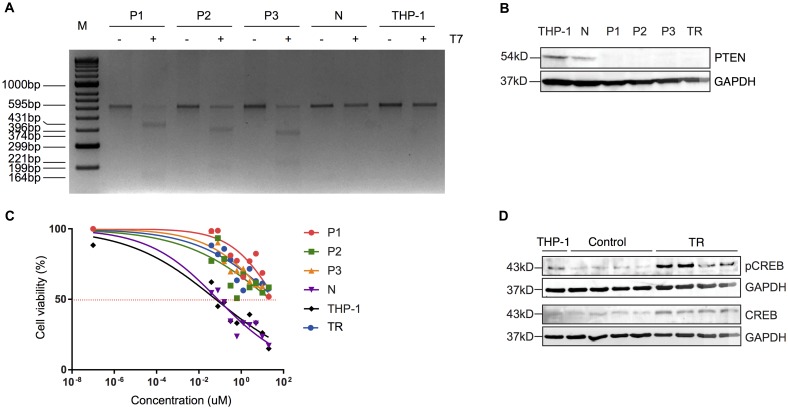
Truncated PTEN is associated with resistance to MEKi via increased activity of CREB. (**A**) T7 endonuclease I assay of the CRISPRed THP-1 lines (P1, P2, P3 and N). (**B**) Representative Western blot of PTEN (54 kD) in each of the CRISPRed lines (N, P1, P2, P3), parental THP-1 cells and TR cells. GAPDH (37 kD) was used as a loading control. (**C**) Sensitivity of CRISPRed lines to AZD6244. Four independent experiments were performed and the mean of each sample at a given AZD6244 concentration was plotted. (**D**) Representative Western blot of pCREB and tCREB in the parental cells, control cells (1–4) and resistant cells (5–8). GAPDH (37 kD) was used as a loading control for each blot.

Although PTEN loss has been suggested to play a role in drug resistance, there has not been any evidence to show the direct link between PTEN loss and drug resistance, particularly in AML, to our knowledge. To investigate whether loss of functional PTEN directly contributes to resistance to MEKi, we used CRISPR/Cas9 technology to introduce the functional loss of PTEN that we observed in resistance cells into parental (and therefore MEKi-sensitive) THP-1 cells. Using a ribonuclear protein (RNP) complex comprised of each of 3 gRNAs that targets the exon 5 of *PTEN* (gP1, gP2, gP3), a fluorophore-labelled scaffolding RNA (tracrRNA) and a recombinant Cas9 protein, we introduced mutations at the locus that encodes the exon 5 of *PTEN.* These mutations resulted in a frameshift in the translation of the protein, as described previously [26], resulting in abrogation of the core catalytic motif in PTEN (Supplementary Figure 3A). An RNP complex using a gRNA that targets a gene desert region (gN) was included as a control for CRISPR. The 3 *PTEN*-targeting gRNAs were tested using a T7 endonuclease I assay, and all were proven to be able to introduce mutations into the designated locus in cells named P1, P2, P3 (Supplementary Figure 3B, 3C). Cells CRISPRed with gN (N) did not show any specific cleavage at this locus (Supplementary Figure 3C). Interestingly, CRISPRed cells (P1, P2, P3 and N) were viable when we expanded each of them 24 h post transfection in culture to a density of 10^6^ cells/ml. Further T7 endonuclease I assays on P1, P2, P3, N and THP-1 cells revealed that mutations at the *PTEN* locus were retained, and THP-1 and N cells were not digested by T7, indicating that N cells share the same genomic sequence as the parental cells at the exon 5 of *PTEN* (Figure 3A). These results were further supported by Western blots, where THP-1 and N cells had comparable levels of PTEN (Figure 3B). In contrast, PTEN was undetectable in P1, P2 and P3 cells, and in a previously established resistant line (TR) (Figure 3B). We then measured IC_50_ of P1, P2, P3, and N cells, along with the THP-1 cells and TR cells, in the presence of AZD6244. We found that the N cells had a similar IC_50_ to THP-1 cells (IC_50_~0.2 uM) while P1, P2, and P3 all had IC_50_s similar to TR cells (IC_50_ >20 uM) (Figure 3C). These results showed that the off-target effect of CRISPR had minimal impact on THP-1 sensitivity to AZD6244, and, for the first time, shows the direct contribution of functional PTEN loss to the establishment of MEKi resistance.

### PTEN loss correlates with an increase in transcription and activation of CREB

It has reported that CREB is a direct target of PTEN where PTEN physically interacts with, and dephosphorylates CREB at Serine 133 [27]. Interestingly, cancerous cells have been shown to restore the CREB phosphorylation that is suppressed upon MEK inhibitor treatment [28]. Therefore, we speculated that loss of the functional domain of PTEN increases CREB phosphorylation (pCREB) in leukemic cells, thus promoting cell survival and MEKi resistance. To test this hypothesis, we measured the total CREB (tCREB) levels and pCREB levels (S133) in TR, control and parental (THP-1) cells. Our results showed that parental cells and control cells had comparably low levels of tCREB and pCREB, while resistant cells showed elevated levels of both tCREB and pCREB (Supplementary Figure 4). This result suggests that loss of the functional domains of PTEN, at least in part, gives rise to an increased phosphorylation of CREB at Ser133, which may promote MEKi resistance as previously reported (Figure 3D) [28].

## DISCUSSION

An improved treatment strategy is desperately needed for pediatric AML due to the side effects of current chemotherapy regimens. The Ras/Raf/MEK/ERK1/2 pathway plays an essential role in human cancers and its contribution in leukemogenesis has been supported by a high incidence of mutations identified in the *RAS* gene family, such as *NRAS* [29–31] and by inappropriate activation of the pathway. MEK inhibitors are small molecules that have shown promise in clinical trials. However, little is known about their therapeutic potential in treating pediatric AML or whether mutations in *RAS* gene family could predict sensitivity to MEK inhibitors. Therefore, we used seven MEK inhibitors that have been investigated in clinical trials and examined their efficacy against a panel of 11 AML cell lines, six of which were pediatric (Table 1).

Our data demonstrate that this response to MEK inhibitors is independent of *RAS* mutational status. THP-1 cells (NRAS^G12D^) exhibited nanomolar IC_50_s for six of the seven MEKi. In contrast, AML-193 cells (NRAS^G13V^) exhibited nanomolar sensitivity to just four of the seven MEKi. However, other cell lines such as MV-4-11 and OCI-AML3 that do not express a *RAS* mutation exhibit nanomolar IC_50_ values in six of the seven MEKi. This resistance may be explained by other oncogenic events in AML that drive Ras pathway activation such as *FLT3*-ITD mutations. Indeed, MV-4-11 possesses this mutation. This observation has been supported by extensive clinical studies in solid tumours such as melanoma where the presence of *Ras/Raf* mutations did not elicit a uniform response and the variations were considered to be a result of co-occurring mutations [32, 33]. A recent study with AZD6244 in AML, however, showed that no patient with *FLT3*-ITD responded to treatment, whilst 17% of *FLT3*-WT patients exhibited a response [13]. Taken together, these data indicate that the sensitivity to MEKi is not likely to be easily stratified according to a single mutation, including *Ras* or *Raf* mutations.

Interestingly, two cell lines (CMK and HEL) exhibited overt intrinsic resistance to MEKi with IC_50_ above 20 μM (Table 1). These cell lines share the common element of a mutation in the JAK/STAT pathway and a p53^M133K^ mutation. CMK cells express a JAK3^A572V^ mutation and HEL express the well-characterized JAK2^V617F^ mutation. Whilst numerous reports suggest activation of the PI3K/AKT pathway may elicit resistance to MEKi due to the nature of cross-talk between these two signaling pathways [34], the role of JAK/STAT pathway activation has not been extensively explored. Future studies investigating the functional influence of JAK/STAT signaling on MEKi sensitivity may have an important clinical impact for classifying patients who are unlikely to respond to MEKi monotherapy, or those where addition of a JAK inhibitor, such as ruxolitinib or pacritinib, may prove beneficial.

Despite the maintenance of a common structural backbone in all MEK inhibitors, a range of anti-proliferative efficacy was observed, suggesting that a subset of AML cells are likely to require co-treatment with a second compound to induce complete population death. This hypothesis has been supported by *in vitro* studies and clinical trials of MEKi [35, 36]. We showed that combinations of MEKi with the cytotoxic agents, cytarabine and daunorubicin - currently used in chemotherapy, appeared to be overall synergistic. However, combinations with etoposide varied depending on the MEKi and the cell line (Table 2). It should be noted that all past and current clinical trials investigating the use of MEKi in AML have been in adults and, as such, the preclinical data presented here provide a rationale for investigating the use of chemotherapy supplemented with MEKi in pediatric AML patients.

Increasing amounts of effort have been spent on exploring how cancer cells acquire resistance to MEK inhibitors. We attempted to investigate the genetic attributes of MEKi resistance. After establishment of MEKi resistance in independent populations of an AML cell line we observed only one universally occurring mutation – copy loss of a functional domain in *PTEN* (Figure 1). No other acquired point mutations or copy changes (Supplementary File 2) in the targeted exome panel were observed in all resistant replicates compared to the control replicates. This mutation appeared to be clonally dominant suggesting that it was able to confer a significant survival advantage on these cells while being challenged with a MEKi and also that it likely occurred prior to the other acquired mutations that we observed. Furthermore, we were able to demonstrate that abrogation of PTEN function by introducing what is effectively the same acquired mutation into the parental, MEKi-sensitive cell line was sufficient to confer MEKi resistance in these cells. It is possible that other mutations were universally acquired in the resistant replicates in other regions of the genome as we interrogated only the genes present in the targeted exome panel. However, the evidence that the single CRISPRed *PTEN* mutation was able to confer the same level of acquired MEKi resistance on the parental cells is highly suggestive that this single event is sufficient for resistance given the genetic background of this AML cell line. Similarly, when we treated these cells with other MEKi, we again observed a lack of sensitivity to these agents suggesting that there is a common mechanism of resistance (Table 3). Interestingly, *PTEN* loss is associated with resistance to MEK inhibitors in a variety of cancers, and in fact may be a common mechanism for conferring resistance to other therapies such as PI3Kβ and BRAF inhibitors and immunotherapy [21, 22, 37–39].

**Table 3:** MEK inhibitor resistance in THP-1 AML cells

Cell line	AZD6244	MEK162	GSK1120212	AS703026
**Parental**	0.3	0.43	0.08	0.23
**TR**	>20	>20	>20	>20

Values shown are IC_50s,_ defined as the concentration of drug that reduces cE by non-linear regression. Values >20 uM indicate where an exact IC50 coulc Ara-C; cytarabine arabinoside.

In addition to PTEN loss, we observed some additional universal copy changes (Figure 1). Because these copy change events appear to be present in a smaller proportion of cells than the *PTEN* loss we believe these changes occurred subsequent to the *PTEN* event and may be secondary to acquired MEKi resistance. For example, we observed amplification of *KRAS.* While the role of mutated *KRAS* in cancer and therapy resistance has been widely described, *KRAS* gene amplification appears to be associated with tumour phenotype rather than with the acquisition of therapy resistance [40]. Loss of *IRF4*, an event we observed in all of our resistant replicates, is reported to be associated with leukemic progression in both lymphoid and myeloid cells, which is consistent with the tumour suppression function of this protein [41–43]. Finally, we observed universal loss of *ELOVL2* in our resistant replicates. While this gene has been implicated in cancer [44, 46] it is unclear what role this event may play in either leukemia or therapy resistance.

Interestingly, three resistant replicates acquired a single nucleotide variant in *NTRK2* at the same position (chr9:87570296) potentially causing a G>D substitution at amino acid 679 in the protein (Supplementary Figure 2). This mutation has not been previously reported so it is unclear what affect, if any, it may have on protein function. One study found that NTRK2 activation cooperates with PTEN loss in T-ALL [45, 47] so it is tantalizing to hypothesize that this mutation acts in a similar way in these cells providing them with yet another survival advantage. Indeed, the observed variant is only three residues from the active site of this enzyme and is proximal to autophosphorylated tyrosine residues (based on a mapping of UniProt entry Q16620 to protein structure 1WWB.X) [39].

While *PTEN* loss has been previously implicated in resistance to a number of other therapeutic agents, this is the first time that it has been shown directly and in AML. We were also able to demonstrate that MEKi synergize with other chemotherapeutic compounds, at least in some cases, so, while MEKi utility in AML is clearly limited by the development of resistant subclones, combination therapy may be a viable strategy to mitigate this effect. In addition, establishment of *PTEN* status at diagnosis, as well as the status of other events known to contribute to MEK signaling-independent growth (e. g., *FLT3*-ITD) may identify patients that are unlikely to respond to MEKi therapy and thus inform alternative treatment approaches.

## MATERIALS AND METHODS

### Cell culture and reagents

All human AML cell lines were purchased directly from recognized repositories; Kasumi-1, MV-4-11, CMK, AML-193, M-07e, HL-60, ML-2, OCI-AML3, ME-1 and HEL from DSMZ (Braunschweig, Germany) and THP-1 from ATCC (Manassas, USA).

AZD6244, PD0325901, GSK1120212, CI-1040, TAK-733, AS703026, MEK162, cytarabine (cytosine arabinoside), daunorubicin hydrochloride (daunorubicin) and etoposide were obtained from Selleck chemicals (Houston, USA). ARRY-162 was purchased from ChemieTek (Indiana, USA). All compounds were dissolved in DMSO and stored at –20° C.

### Cell proliferation, cell cycle and apoptosis assays

Human AML cell lines (2 × 10^4^ cells/well) were seeded in a 96-well plate with appropriate factors and the indicated concentrations of compound for 96 hours. Assays were plated in quadruplicate and repeated at least three times. Proliferation was assessed using a resazurin reduction assay (CellTiter-Blue™, Promega, WI, USA). The concentration of compound that reduced cell viability by 50% (IC_50_) was determined using non-linear regression with variable slope after normalising fluorescence to untreated cellular controls. For combination assays, cells were treated with fixed ratio concentrations of one MEKi (GSK1120212, AS703026 or AZD6244) in combination with one cytotoxic compound (cytarabine, daunorubicin or etoposide) for 96 h. For cell cycle analysis, cells (2 ×10^5^ cells/mL) were seeded in 96-well plates with appropriate factors and the indicated compound concentrations for 96 h. Assayed cells were fixed in 100 μL 70% ethanol in PBS at 4° C. Cells were washed in PBS then incubated with 40 mg/mL propidium iodide (PI; Sigma Aldrich, MO, USA) and 250 mg/mL RNase (Sigma Aldrich) for 30 minutes at 37° C. Apoptosis was measured using the annexin-V FITC apoptosis detection kit according to manufacturer’s instructions (BD Biosciences). AML cell lines were seeded at 2 × 10^5^ cells/mL and treated with indicated concentrations of compound for 96 h. Samples were stained for annexin-V FITC (BioLegend, CA, USA) and PI. Samples were analyzed on an LSRII Fortessa^TM^ flow cytometer (BD Biosciences, NJ, USA), data were acquired using the BD FACSDiva software and analysed with FlowJo software.

### Immunoblotting

Cells were washed and lysed as previously described [23]. Total protein (10μg) was separated by SDS-PAGE and transferred onto nitrocellulose membranes (GE Healthcare, Buckinghamshire, England). Antibodies used for immunoblotting included anti-pMEK1/2(Ser217/221), -MEK1/2 total, -ERK1/2(Thr202/Tyr204), -ERK1/2 total, PARP, -pAKT (S473), -AKT, pCREB (S133) and CREB (Cell Signalling Technology, Inc., MA, USA) and anti-GAPDH (Millipore, USA). Membranes were incubated with IRDye^®^ 680LT or IRDye 800CW conjugated secondary antibodies and protein-antibody complexes visualized as previously described [48].

### Development of MEKi-resistant THP-1 cells

THP-1 cells with resistance to AZD6244 (referred to as TR) were developed by culturing THP-1 cells in the presence of increasing concentrations of the compound (with ≤0.1% DMSO) until confluent growth was sustained in 20 times the IC_50_ (>10 μM). Resistance was confirmed by incubating cells in drug free media for a minimum of one week followed by CTB assay with drug concentrations up to 20 μM. Subsequent experiments using the TR lines were carried out on cells incubated in drug free media for a minimum of one week. The resistance development was replicated 8 independent times and paired with DMSO treated controls to establish background mutation rate occurring over time with culture.

### Exome sequencing

Libraries were prepared using the Nugen Ovation Target Enrichment System and 2 × 150 bp paired end sequencing was performed on the Illumina MiSeq v2 at the Monash Health Translation Precinct Medical Genomics Facility. The Ovation Cancer Panel 2.0 Target Enrichment Probes panel was used to perform targeted exome sequencing of known cancer genes to mean depth of coverage of 53× (range 47–67×) according to manufacturer’s specifications. Briefly, 500ng of DNA from 8 resistant cell lines and the paired DMSO controls was sheared, ligated to adapter probe and purified. Libraries were pooled for enrichment at equimolar ratios and 15 pM of a single pool was used for clustering. The total run yield was 4.3G encompassing a total of 14,319,766 reads. The 1% PhiX spike in parameters were rated excellent for having an error rate of 0.27% and phasing/prephasing of 0.092/0.051. Reads were aligned to the hs37d5 human reference genome using NovoAlign followed by duplicate read marking and base quality recalibration according to GATK best practices. Single nucleotide variants and small indels were called using mutect2 [49]. To investigate copy number variation, bedtools 2.26.0(CITE: https://academic.oup.com/bioinformatics/article/26/6/841/244688/BEDTools-a-flexible-suite-of-utilities-for) was used to calculate mean coverage depth per exon in each sample. Exons with group mean coverage below 20 reads in both control and treated groups were excluded from the analyses to reduce noise. Read depths in each sample were normalized by total library size, and control and treated normalized exon read depths compared using a Student’s *t*-test (p-values adjusted using the Bonferroni correction) to identify alterations in copy number.

### qPCR validation of *PTEN* loss by transcription quantification

RNA (100ng) was reverse transcribed in a 20 ul reaction using SensiFast cDNA synthesis kit (Bioline, USA). The cDNA was diluted 1 in 10 and 4 ul was used in qPCR with 1 x SYBR green MasterMix (Life Technologies, Thermofisher Scientific, USA) and a pair of gene-specific primers (Supplementary Table 1). Cycling was performed using ViiA 7 thermocycler (Applied Biosystems, USA) with its default cycling conditions. *GUSB* was used as a housekeeper to quantify the *PTEN* expression in samples. Analysis was performed using the 2^−∆∆CT^ method.

### Introducing mutations in the exon 5 of *PTEN*

CRISPR was performed using Ribonuclear protein (RNP) complex system to introduce mutations in the exon 5 of *PTEN*, which encodes the catalytic core motif of the protein [20]. Guide RNAs (gRNAs) were designed using a web designing tool (http://portals.broadinstitute.org/gpp/public/analysis-tools/sgrna-design). A total of 3 gRNAs (gP1, gP2, gP3) that target the bases before the core motif-encoding region were selected based on their target efficiency score (Supplementary Table 2). Each of the 3 gRNAs was then complexed with an ATTO-550 labelled scaffolding RNA (tracrRNA) (IDT, USA) and a recombinant Cas9 protein (NEB, USA) following manufacturer’s instructions. The RNP complexes (10nM) were transfected into THP-1 cells using RNAiMax (Life Technologies, USA); and the cells were cultured at 37° C with 5% CO_2_. A CRISPR experiment using a negative control gRNA (N) (IDT, USA) was also performed along with other CRISPR experiments. Genome editing efficiency was measured 48 h post-transfection.

### Measurement of genome-editing efficiency

Genome editing efficiency was measured using T7 endonuclease I according to the manufacturer’s instructions (NEB, USA). Briefly, genomic DNA (gDNA) was extracted using Genomic DNA isolation kit (Bioline, USA). A pair of primers were used in the presence of the Platinum Taq DNA Polymerase High Fidelity (Life Technologies, USA) to amplify a 595 bp region including 357 bp of the intron 4 that is sitting immediately upstream of the exon 5 and 238 bp of the exon 5 from 200ng of gDNA (Supplementary Table 1). Cycling was performed using Eppendorff PCR thermocycler for a total of 35 cycles. Each cycle started with denaturing at 94° C for 15s, followed by annealing at 60° C for 30s and an extension at 68° C for 30s. The PCR products were then ramped following the manufacturer’s instruction (IDT, USA) and digested using 10 units of T7 endonuclease I at 37° C for 15 min. Fragmentation was separated by gel electrophoresis and visualized by Quantum ST4 Xpress system (Montreal Biotech, USA).

## SUPPLEMENTARY MATERIALS






