# Erratum: *PTEN* deletion drives acute myeloid leukemia resistance to MEK inhibitors

**DOI:** 10.18632/oncotarget.27402

**Published:** 2020-01-21

**Authors:** Amanda M. Smith, Christine R.C. Zhang, Alexandre S. Cristino, John P. Grady, J. Lynn Fink, Andrew S. Moore

**Affiliations:** ^1^ The University of Queensland Diamantina Institute, The University of Queensland, Woolloongabba, Australia; ^2^ Oncology Services Group, Queensland Children’s Hospital, South Brisbane, Australia; ^3^ Child Health Research Centre, The University of Queensland, South Brisbane, Australia; ^4^ Current address: Washington University in Saint Louis, Saint Louis, Missouri, United States of America; ^5^ Current address: Garvan Institute of Medical Research, Darlinghurst, Australia; ^6^ Current address: Griffith Institute for Drug Discovery, Brisbane Innovation Park, Nathan, Australia

**This article has been corrected:** During production, the 4th affiliation was mistakenly added to the name of the last author, Dr. Andrew S. Moore. The proper affiliation information is shown below.

Original article: Oncotarget. 2019; 10:5755–5767. 5755-5767. https://doi.org/10.18632/oncotarget.27206

Amanda M. Smith^1,4,*^, Christine R.C. Zhang^1,4,*^, Alexandre S. Cristino^1,6^, John P. Grady^1,5^, J. Lynn Fink^1^ and Andrew S. Moore^1,2,3^

^1^The University of Queensland Diamantina Institute, The University of Queensland, Woolloongabba, Australia

^2^Oncology Services Group, Queensland Children’s Hospital, South Brisbane, Australia

^3^Child Health Research Centre, The University of Queensland, South Brisbane, Australia

^4^Current address: Washington University in Saint Louis, Saint Louis, Missouri, United States of America

^5^Current address: Garvan Institute of Medical Research, Darlinghurst, Australia

^6^Current address: Griffith Institute for Drug Discovery, Brisbane Innovation Park, Nathan, Australia

^*^These authors contributed equally to this work

