# Supplementary material for: PTEN deletion drives acute myeloid leukemia resistance to MEK inhibitors
Source: Oncotarget. 2019 Oct 8;10(56):5755–67. doi: 10.18632/oncotarget.27206 (PMC6791388; doi:10.18632/oncotarget.27206)
Supplement: Supplementary file 1 [file oncotarget-10-5755-s001.pdf]

PTEN deletion drives acute myeloid leukemia resistance to MEK inhibitors

SUPPLEMENTARY MATERIALS

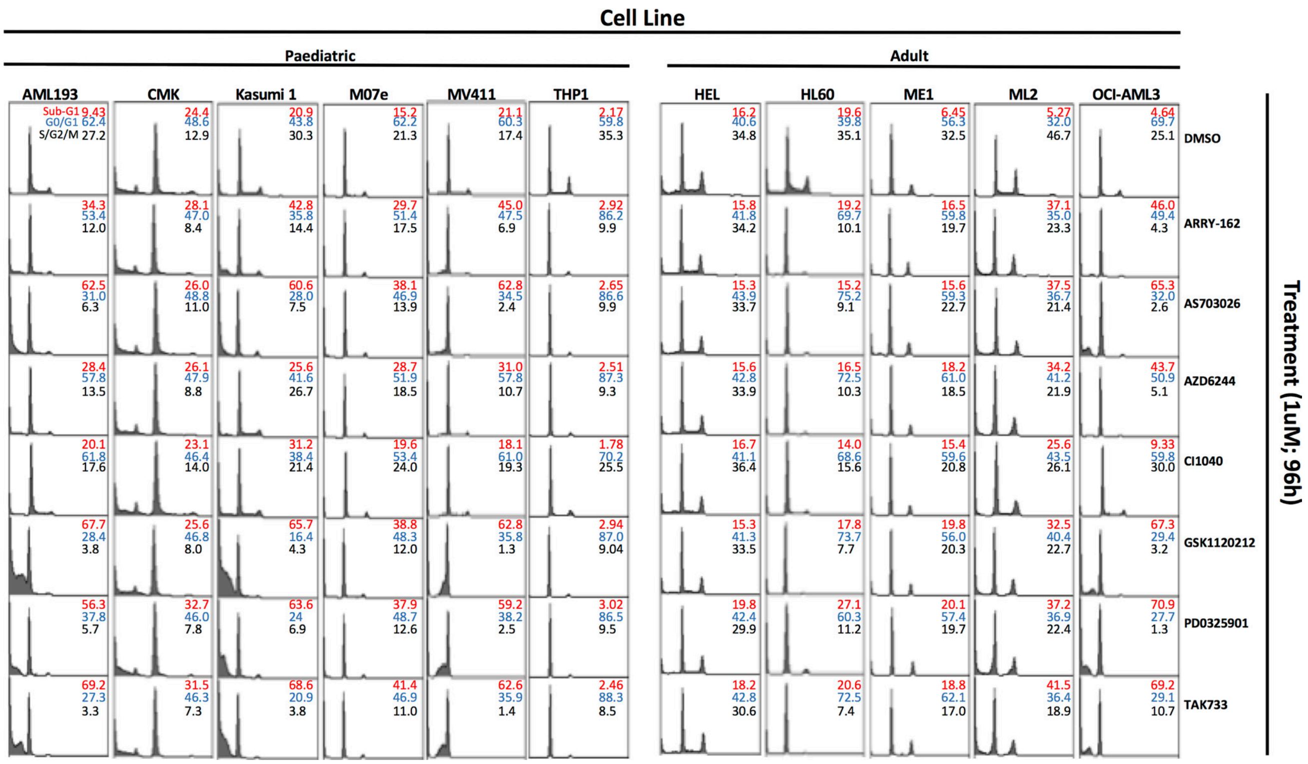

Supplementary Figure 1: MEK inhibitors induce cytostatic and cytotoxic effects in AML. AML cell lines were treated with MEK inhibitors (1 µM; 96 h) or DMSO equivalent, stained with propidium iodide and cell cycle was assessed by flow cytometric analysis. Populations were gated on FSC-H vs FSC-W to select for singlets. Red indicates the percentage of sub-G1, blue indicates percentage of G0/G1 and black indicates the percentage of S/G2/M. Histograms are representative examples from 1–6 replicates.

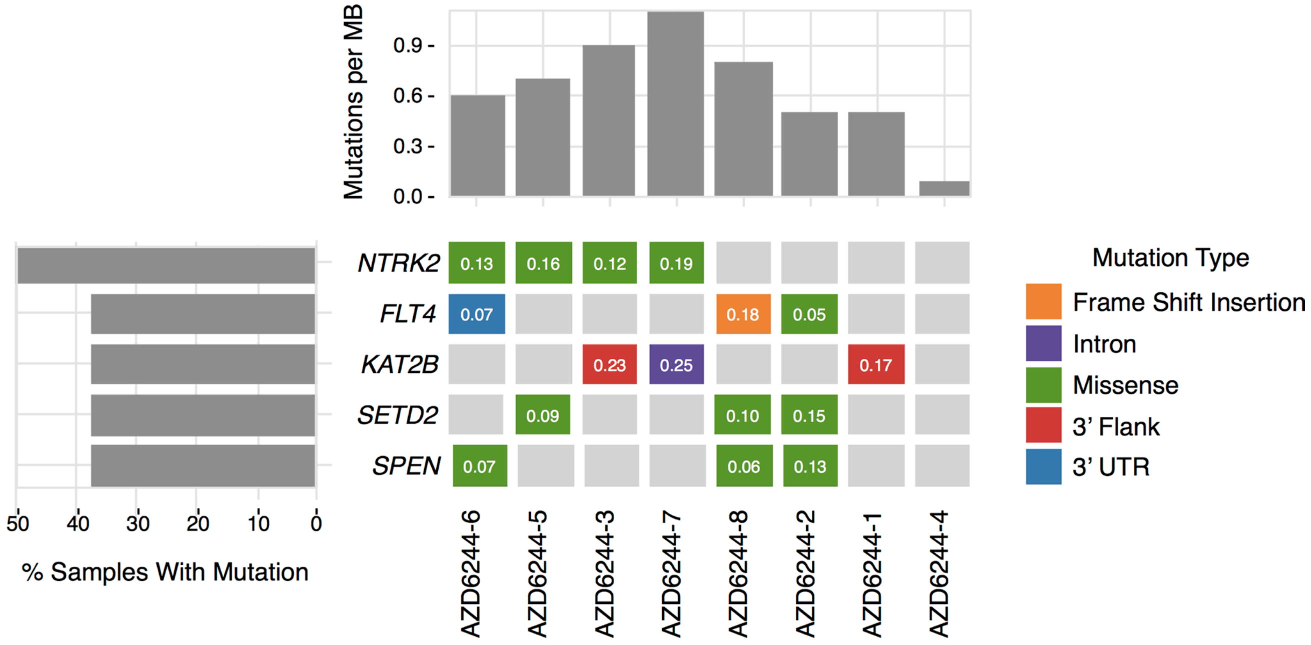

Supplementary Figure 2: Single nucleotide variants acquired during generation of MEKi-resistant phenotype. Single nucleotide variant calling of all TR populations compared to their DMSO control showed that no SNV or mutated gene was common to all replicates. Variant allele frequencies are shown inside the coloured boxes.

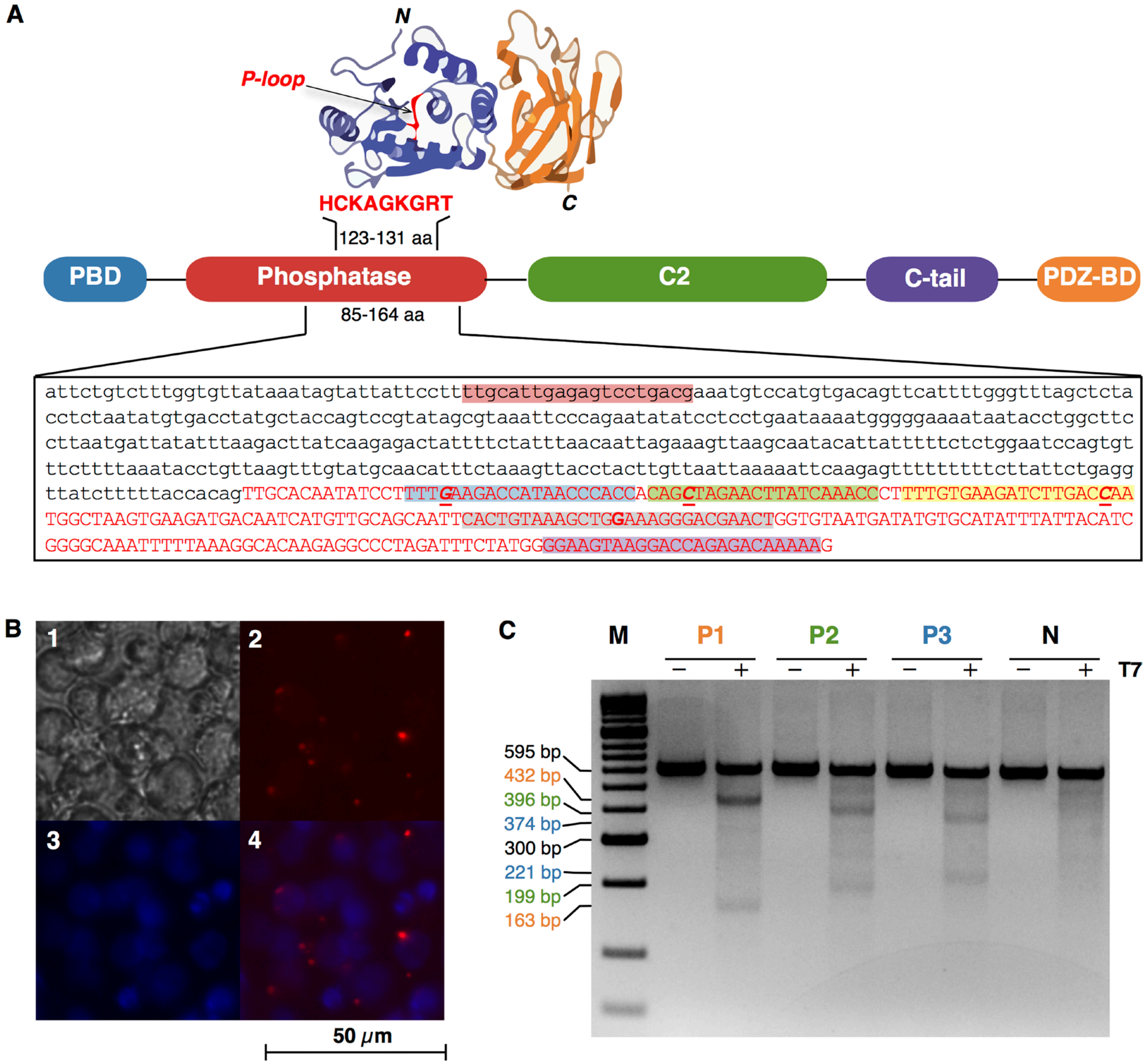

Supplementary Figure 3: Introducing mutations in exon 5 of *PTEN*. (**A**) A predicted PTEN domain structure was adapted from [48], which shows the core catalytic motif of PTEN (P-loop, in red). This motif is encoded by the 123 -131 aa "HCKAGKGRT" sequence which is part of exon 5 (85-164 aa). The DNA sequences of exon 5 (red capital letters) and 374 bp of the intron 4 (black lowercase letters) are shown in the box. Primer sequences used for T7 endonuclease I assay (595 bp) are highlighted in pink and purple. The gRNAs, gP1, gP2, and gP3, are highlighted in yellow, green, and, blue, respectively. Grey shows the core-motif encoding sequence. (**B**) 48 h post-transfection of RNP complex in THP-1 cells. Brightfield, cy3 and DAPI channels were shown in 1), 2), and 3). 4) depicts a combined channel of cy3 and DAPI and shows that RNP complexes overlap with chromosomes, indicating genome-editing occurred. (**C**) Gel photo of T7 endonuclease I assay of cells CRISPRed by gP1 (P1), gP2 (P2), gP3 (P3) and gN (N). The uncut PCR products (-) were electrophoresed with the T7-digested PCR products (+). P1 (+) yielded additional two fragments 432 bp and 163 bp; P2 (+) showed 396 bp and 199 bp; P3 (+) yielded 374 bp and 221 bp. N (+) did not have any bands similar to P1, P2, or P3.

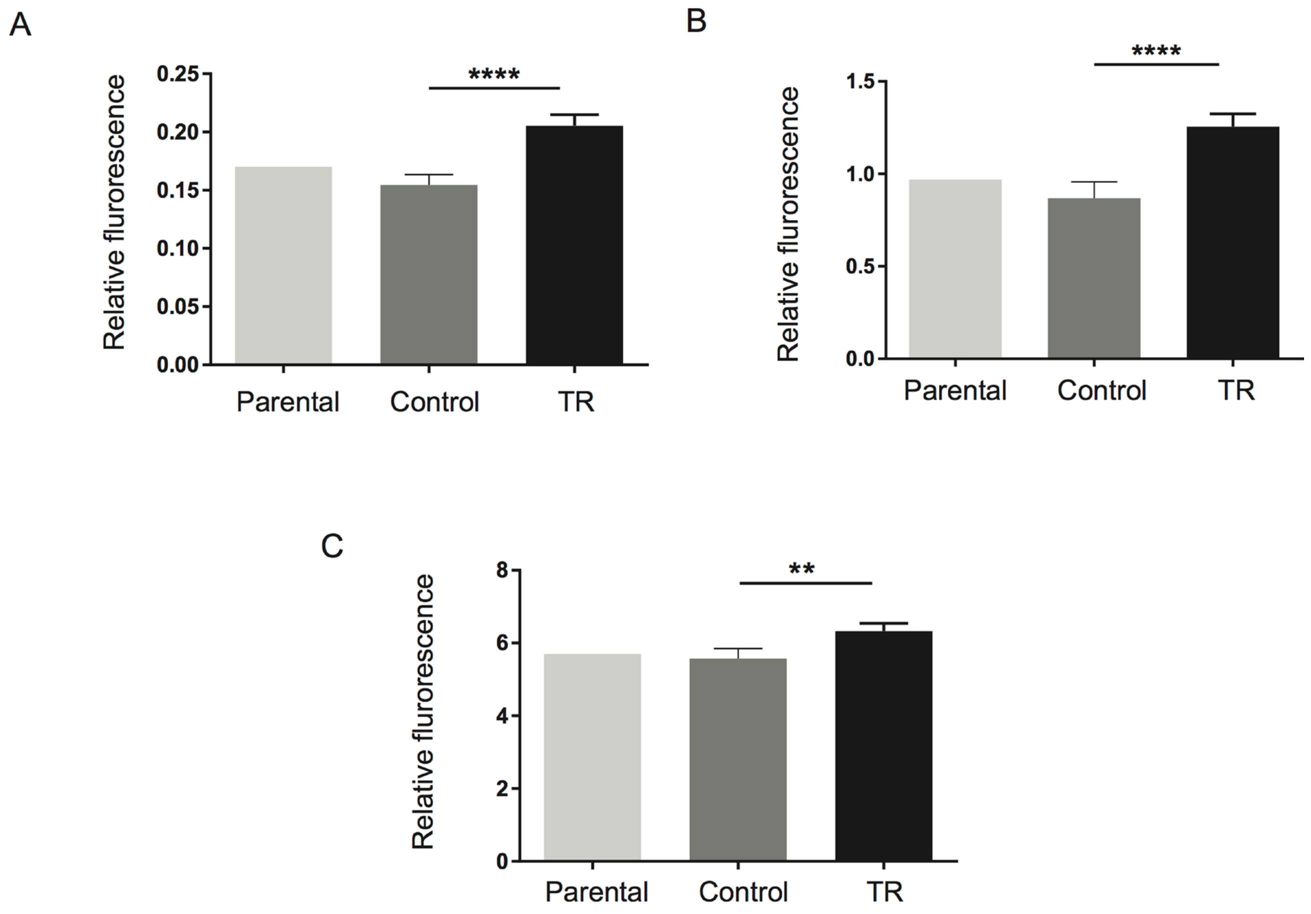

Supplementary Figure 4: Quantification of pCREB A) and tCREB B) levels in parental cells, control cells and resistant cells. The ratio between pCREB and tCREB has been shown in C. Data are presented as mean and standard deviation. $^{****}p < 0.0001$; $^{**}p < 0.01$ (*t*-test, two-tailed).

Supplementary Table 1: Primers for quantification of *PTEN* expression

Region	Direction	Sequence (5′-3′)
Exon 1-2	F	TGACAGCCATCATCAAAGAGA
	R	CAATATTGTTCCTGTATACGCCTTC
Exon 5	F	TGGCTAAGTGAAGATGACAATCA
	R	TTTTTGTCTCTGGTCCTTACTTCC
Exon 8-9	F	GCGTGCAGATAATGACAAGG
	R	GCTAGCCTCTGGATTTGACG

Supplementary Table 2: Guide RNAs targeting *PTEN* exon 5

Name	Orientation	sgRNA Cut Position (1-based)	sgRNA Sequence	sgRNA Context Sequence	PAM Sequence	On-Target Efficacy Score
gP1	Sense	75	TTTGTGAAGATCTTGACCAA	CCCTTTTGTGAAGATCTTGACCAATGGCTA	TGG	0.6379
gP2	Antisense	39	GGTTTGATAAGTTCTAGCTG	AAAGGGTTTGATAAGTTCTAGCTGTGGTGG	TGG	0.6482
gP3	Antisense	18	GGTGGGTTATGGTCTTCAAA	CTGTGGTGGGTTATGGTCTTCAAAAGGATA	AGG	0.4794

Supplementary File 1: DMSO and TR cell line single nucleotide variants. See Supplementary File 1

AZD6244_targeted_exome_SNVs. maf

Supplementary File 2. DMSO and TR cell line exon coverage depths. See Supplementary File 2

AZD6244_targeted_exome_exon_depths. txt
